# Lipoxin A_4_ Stimulates Calcium-Activated Chloride Currents and Increases Airway Surface Liquid Height in Normal and Cystic Fibrosis Airway Epithelia

**DOI:** 10.1371/journal.pone.0037746

**Published:** 2012-05-25

**Authors:** Valia Verrière, Gerard Higgins, Mazen Al-Alawi, Richard W. Costello, Paul McNally, Raphaël Chiron, Brian J. Harvey, Valérie Urbach

**Affiliations:** 1 Department of Molecular Medicine, RCSI Education and Research Centre, Beaumont Hospital, Dublin, Ireland; 2 CRCM, Montpellier, France; 3 Department of Respiratory Medicine, Beaumont Hospital, Dublin, Ireland; 4 National Children’s Research Centre, Our Lady’s Children’s Hospital Crumlin, Dublin, Ireland; Abramson Research Center, United States of America

## Abstract

Cystic Fibrosis (CF) is a genetic disease characterised by a deficit in epithelial Cl^−^ secretion which in the lung leads to airway dehydration and a reduced Airway Surface Liquid (ASL) height. The endogenous lipoxin LXA_4_ is a member of the newly identified eicosanoids playing a key role in ending the inflammatory process. Levels of LXA_4_ are reported to be decreased in the airways of patients with CF. We have previously shown that in normal human bronchial epithelial cells, LXA_4_ produced a rapid and transient increase in intracellular Ca^2+^. We have investigated, the effect of LXA_4_ on Cl^−^ secretion and the functional consequences on ASL generation in bronchial epithelial cells obtained from CF and non-CF patient biopsies and in bronchial epithelial cell lines. We found that LXA_4_ stimulated a rapid intracellular Ca^2+^ increase in all of the different CF bronchial epithelial cells tested. In non-CF and CF bronchial epithelia, LXA_4_ stimulated whole-cell Cl^−^ currents which were inhibited by NPPB (calcium-activated Cl^−^ channel inhibitor), BAPTA-AM (chelator of intracellular Ca^2+^) but not by CFTRinh-172 (CFTR inhibitor). We found, using confocal imaging, that LXA_4_ increased the ASL height in non-CF and in CF airway bronchial epithelia. The LXA_4_ effect on ASL height was sensitive to bumetanide, an inhibitor of transepithelial Cl^−^ secretion. The LXA_4_ stimulation of intracellular Ca^2+^, whole-cell Cl^−^ currents, conductances and ASL height were inhibited by Boc-2, a specific antagonist of the ALX/FPR2 receptor. Our results provide, for the first time, evidence for a novel role of LXA_4_ in the stimulation of intracellular Ca^2+^ signalling leading to Ca^2+^-activated Cl^−^ secretion and enhanced ASL height in non-CF and CF bronchial epithelia.

## Introduction

Cystic fibrosis is caused by the mutation of the gene coding for the Cystic Fibrosis Transmembrane conductance Regulator (CFTR), a cyclic AMP-dependent Cl^−^ channel. The major clinical features of CF are chronic pulmonary disease, exocrine pancreatic insufficiency and male infertility [1], [2]. The lung disease is the main cause of morbidity and mortality in CF. The airway epithelium of patients with CF fails to transport Cl^−^ and water, resulting in a reduced ASL height and impaired mucociliary clearance. The hyper-absorption of Na^+^ observed in the CF bronchial epithelium may further exacerbate the dehydration of the ASL. It is generally accepted that the dehydration of the airway lumen favours chronic infection and inflammation leading to progressive destruction of the lung [3]. Identification of agents, particularly natural endogenous biologicals, which stimulate alternative non-CFTR Cl^−^ secretory pathways and promote ASL hydration and recovery of optimal ASL height are likely to be of therapeutic benefit in improving mucociliary clearance in patients with CF.

The levels of LXA_4_ in the airways have been reported to be decreased in patients with CF [4]. Lipoxins are bioactive lipids derived from omega-6 polyunsaturated fatty acids and play important roles in various biological functions [5]. The endogenous lipoxin A_4_ (LXA_4_: 5S,6R,15S-trihydroxy-7,9,13-trans-11-eicosatetraenoic acid) is produced at inflammatory sites from the interaction of lipoxygenase activities of several cell types including leukocytes, platelets and epithelial cells. LXA_4_is one member of the newly identified lipid molecules playing a role in ending/resolving the inflammatory process by modulating neutrophilic inflammation, clearing apoptotic PMN and inhibiting the production of pro-inflammatory cytokines [6]. The deficit in LXA_4_ levels in CF airways could be a contributing factor in chronic airway inflammation which characterises these patients.

Very little is known about the role of LXA_4_ in the lung beyond its anti-inflammatory effects. We have previously shown that normal human bronchial epithelial cells are a biological target for LXA_4_. The receptor for LXA_4_ (ALX/FPR2) is expressed in the bronchial epithelial cell line 16HBE14o- and LXA_4_ stimulates an intracellular Ca^2+^ mobilisation in these cells [7]. Intracellular Ca^2+^ is a major regulator of Cl^−^ transport and the stimulation of epithelial Cl^−^ secretion would be of major therapeutic benefit in CF to restore efficient airway clearance. We have investigated the effect of LXA_4_ on epithelial Cl^−^ secretion and its functional consequences on ASL height using bronchial epithelial cells obtained from CF and non-CF patient biopsies and in a variety of bronchial epithelial cell lines commonly used as models for CF ion transport and immunological studies.

## Results

### LXA_4_ Effects on Intracellular Ca^2+^ in Normal and CF Bronchial Epithelial Cells

**Figure 1:**
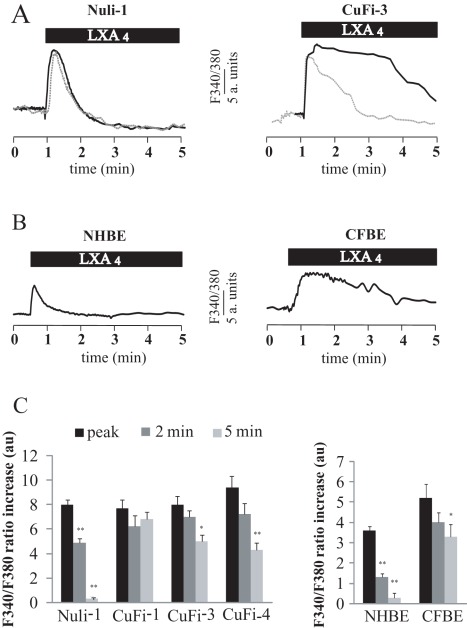
LXA_4_ effect on intracellular Ca^2+^ activity in non-CF and CF bronchial epithelial cells. (A) Typical effect of LXA_4_ (100 nM) on the cytosolic Ca^2+^ (ratio F340/F380) measured in Nuli-1 and CuFi-3 cell lines in control (plain line) and in external Ca^2+^ -free conditions (dotted line). (B) Typical effect of LXA_4_ (100 nM) on the cytosolic Ca^2+^ in normal and CF primary cultures of bronchial epithelial cells (NHBE and CFBE). (C) Mean values of the maximum increase in Ca^2+^ (peak) and measured 2 and 5 minutes after the peak, in Nuli-1 (n = 6), CuFi-1 (n = 6), CuFi-3 (n = 6), CuFi-4 (n = 4) cell lines and in NHBE (n = 4) and CFBE (n = 4) bronchial epithelial cells in primary culture (* p<0.05, ** p<0.01).

LXA_4_ induced a rapid increase of intracellular Ca^2+^ in all human airway epithelial cell types tested. These results are summarised in typical records obtained in Nuli-1 and CuFi-3 cell lines (figure 1A) and NHBE and CFBE primary cultures (Figure 1B) and on corresponding histograms (Figure 1C). There was no significant difference in the maximum increase in calcium induced by LXA_4_ (100 nM) between non-CF and CF bronchial epithelial cell lines (Figure 1A and 1C). However the maximum increase obtained in CF bronchial epithelial primary culture (CFBE) was significantly higher than in non-CF (NHBE) bronchial epithelial primary cultures (Figure 1B and 1C). The kinetics of the Ca^2+^ responses were markedly different between CF and non-CF cells. In the non-CF bronchial epithelial cells (NHBE primary cultures and Nuli-1 cell line), the intracellular Ca^2+^ rise induced by LXA_4_ was fast and transient with a recovery to basal values within 2 to 5 min (Figure 1C). In contrast, in the CF bronchial epithelial cells (CFBE primary cultures and CuFi cell lines), LXA_4_ induced a slower increase in Ca^2+^ and a delayed (or absent) recovery toward basal values (Figure 1C). Thus the total amount of Ca^2+^ mobilised in the cytosol upon LXA_4_ exposure was higher in CF cells than in non-CF bronchial epithelial cells.

In order to investigate the origin of the calcium signal induced by LXA_4_, we tested the effect of LXA_4_ on intracellular Ca^2+^ mobilization in the absence of extracellular Ca^2+^ in non-CF and CF airway epithelial cells. The results presented in Figure 1A show that in Nuli-1 cells bathed in an external Ca^2+^-free solution, the response to LXA_4_ was not different from control conditions (plain line). In Nuli-1 cells, there was no significant difference in the maximum Ca^2+^ increase obtained after LXA_4_ exposure with or without external Ca^2+^ (F340/F380 : control 8.49±0.48 and external Ca^2+^-free 8.57±0.32) and in the value measured 2 min after the peak (5.22±0.43 in control condition and 5.89±0.36 in external Ca^2+^-free (n = 4, p>0.1)). These results indicate that in normal airway epithelial cells, LXA_4_ generates a calcium signal mainly due to the release of Ca^2+^ from intracellular stores rather than Ca^2+^ entry. As shown in Figure 1A, there was no difference in the maximum Ca^2+^ increase in CuFi-3 cells obtained after LXA_4_ exposure with or without external Ca^2+^ (F340/F380: control 8.76±0.55 and external Ca^2+^-free: 8.99±0.49 (n = 4, p>0.1)). However, in external Ca^2+^-free medium, the calcium response to LXA_4_ in CuFi-3 cells was more transient with a rapid recovery to basal values. The F340/F380 ratio values obtained 2 min after the peak Ca^2+^ response were 7.17±0.43 in control conditions and 2.29±0.46 (n = 4, p<0.05) in an external Ca^2+^-free solution. Taken together, these results suggest that, in CF airway epithelia, in addition to the Ca^2+^ mobilisation from intracellular stores, LXA_4_ also stimulates Ca^2+^ entry and this response is absent in non-CF bronchial epithelial cells (Figure 1A).

### Role of the ALX/FPR2 Receptor in the Calcium Response to LXA_4_

**Figure 2:**
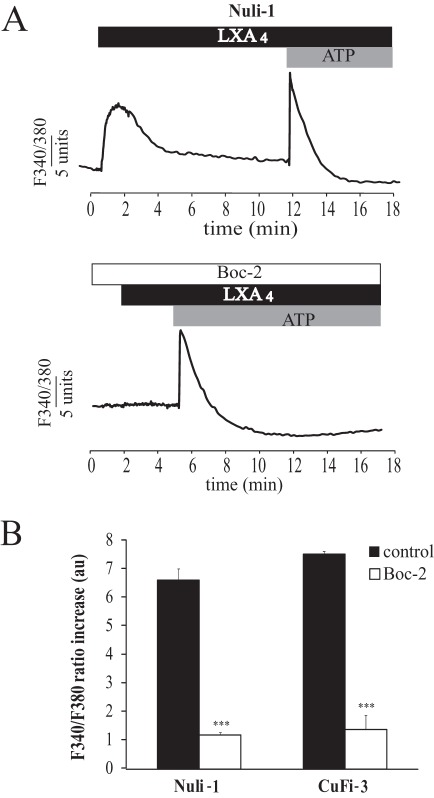
Effect of Boc-2 on the intracellular Ca^2+^ signal induced by LXA_4_. (A) Representative effect of LXA_4_ (100 nM) and ATP (100 µM) on cytosolic Ca^2+^ (ratio F340/F380) in NuLi-1 cells in control conditions (upper panel) and after 24 hours of pre-treatment with Boc-2 (10 µM) a specific inhibitor of ALX/FPR2 (lower panel). (B) Mean values corresponding to the effect of Boc-2 on the Ca^2+^ response to LXA_4_ in Nuli-1 (n = 5) and in CuFi-3 (n = 4) cell lines (*** p<0.001).

The role of the ALX /FPR2 receptor in the Ca^2+^ response to LXA_4_ was investigated using the specific inhibitor, Boc-2 (Figure 2). The effect of LXA_4_ (100 nM) on intracellular Ca^2+^ was completely abolished after treatment with Boc-2 (10 µM) in both NuLi-1 (n = 5) and CuFi-3 (n = 4) cells (figure 2A and 2B). However, ATP (100 µM), a known stimulator of intracellular Ca^2+^ mobilisation via purinergic receptor stimulation, produced a Ca^2+^ signal in cells treated with Boc-2 (Figure 2A). These results support the involvement of the ALX/FPR2 receptor in the Ca^2+^ signalling response to LXA_4_.

### LXA_4_ Effects on Whole-cell Currents in Non-CF and CF Bronchial Epithelial Cells

Since intracellular Ca^2+^ is a regulator of Cl^−^ transport and we have shown that LXA_4_ we regulates Ca^2+^, we investigated the effect of LXA_4_ on ion transport using whole cell patch-clamp recording techniques. The whole-cell current-voltage relationships were obtained from whole-cell patch-clamp recordings in non-CF (NHBE primary culture and Nuli-1 cell line) and CF (CFBE primary culture and CuFi-3 cell line) human bronchial epithelial cells.

**Figure 3:**
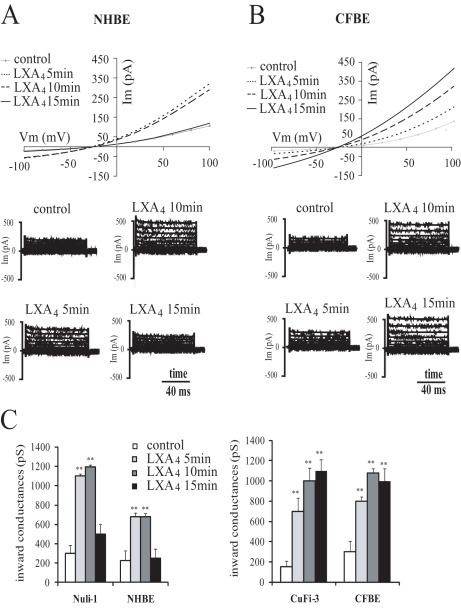
Time dependency of the effect of LXA_4_ on whole-cell currents in normal and CF bronchial epithelial cells in primary culture. Typical I-V relationships and corresponding current records obtained before and after 5, 10 and 15 min exposure to LXA_4_ (100 nM) in NHBE (A) and CFBE (B) isolated bronchial epithelial cells in primary culture. (C) Mean values corresponding to the time dependency of the LXA_4_ effect onwhole cell inward conductances in Nuli-1 (n = 8) and in CuFi-3 (n = 4) cell lines and NHBE (n = 8) and CFBE (n = 6) primary cultures (**p<0.01).

**Figure 4:**
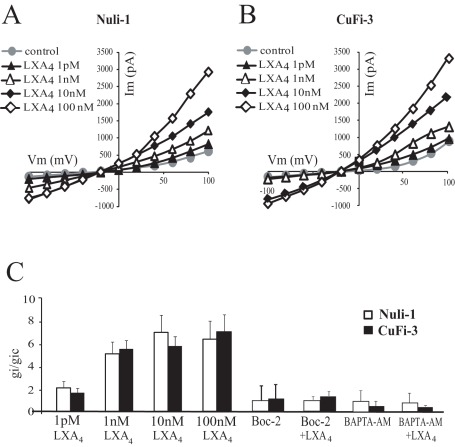
Dose dependency of the effect of LXA_4_ on whole-cell currents of normal (Nuli-1) and CF (CuFi-3) bronchial epithelial cell lines. Typical I-V relationships obtained before and after 10 min exposure to 1 pM (n = 6 Nuli-1, n = 6 CuFi-3), 1 nM (n = 4 Nuli-1, n = 4 CuFi-3), 10 nM (n = 3 Nuli-1, n = 3 CuFi-3) and 100 nM (n = 6 Nuli-1, n = 6 CuFi-3) LXA_4_ in Nuli-1 (A) and CuFi-3 (B) cell lines. (C) Mean inward conductance changes normalized to control values (gi/gic obtained without LXA_4_) as a function of LXA_4_ concentration in Nuli-1 (open bars) and CuFi-3 (black bars) cells in control conditions and obtained upon exposure to Boc-2 (10 µM) alone (10 min, 100 nM, n = 4 Nuli-1, n = 4 CuFi-3) or with Boc-2 (10 µM) and LXA_4_ (10 min, 100 nM, n = 6 Nuli-1, n = 6 CuFi-3) and after BAPTA-AM pre-treatment alone (n = 6 Nuli-1, n = 6 CuFi-3) or with BAPTA-AM and LXA_4_ (10 min, 100 nM, n = 4 Nuli-1, n = 6 CuFi-3).

As shown from the current-voltage curves (Figure 3 and 4) and in Table 1, the whole-cell currents under control conditions were outwardly rectified in non-CF and CF cell lines and in primary airway epithelial cells. The reversal potentials (E_rev_) obtained in the cell lines (E_rev_ Nuli-1 = −23.8±3.2 mV, E_rev_ CuFi-3 = −21. ±3.6 mV) and primary cultures (E_rev_ NHBE = −22.4±3.9 mV and E_rev_ CFBE = −22.4±1.9 mV) indicate that Cl^−^ (E_Cl_ = −39 mV) is the main charge carrier under these conditions (Table 1).

**Table 1:** Mean outward (*G_out_*) and inward (*G_in_*) conductances and reversal potentials (*E_rev_*) measured in non CF and CF airway epithelial cells in primary culture and NuLi-1 and CuFi-3 cell lines in control conditions and stimulated by LXA_4_ (100 nM, 10 min).

		*G_out_ (pS)*	*G_in_ (pS)*	*E_rev_ (mV)*	*n*
**NHBE**	Control	612.2±165.2	227.7±99.3	−22.4±3.9	16
	LXA_4_	1742±147.2	676±36.6	−21.4±5.8	13
**CFBE**	Control	855.4±192.9	302.9±100.4	−22.4±1.9	8
	LXA_4_	3187.5±172.1	1078.14±43.4	−23.8±3.1	8
**NuLi-1**	Control	622.1±168.9	298.7±85.5	−23.8±3.2	8
	LXA_4_	1365.1±75.8	1192±24.2	−22.9±3.7	6
**CuFi-3**	Control	979.6±146.8	150±61.36	−21.7±3.6	5
	LXA_4_	2156.5±232.7	999±132.97	−24.7±4.0	5

LXA_4_ exposure stimulated the whole-cell currents in non-CF and CF airway epithelial cell lines and in normal and CF primary cultures, in a time-dependent and dose-dependent manner, as illustrated in Figure 3 and Figure 4, respectively.

Typical whole-cell current-voltage relationship in NHBE and CFBE primary cultures recorded over variable duration of LXA_4_ (100 nM) exposure illustrate the time-dependence of the response (Figure 3A and 3B). The statistical significance of the time dependence of current responses have been investigated in non-CF and CF airway epithelial cell lines and primary cultures as shown in Figure 3C. The maximum stimulatory effect on the inward conductance (for outward flux of Cl^−^ from the cell) in non-CF airway epithelial cells (Nuli-1 and NHBE) was obtained after 10 min exposure to LXA_4_ and declined thereafter to control levels (Figure 3C). In CF airway epithelial cells (CuFi-3 and CFBE), the increased inward conductance induced by LXA_4_ was sustained without recovery to basal values over the 15 min period of observation (Figure 3C).

The dose dependence of the response to LXA_4_ was investigated in the non-CF and CF cell lines. The stimulatory effect of LXA_4_ on membrane conductance was found to be dose-dependent as illustrated on the typical whole-cell current-voltage relationship obtained from Nuli-1 and CuFi-3 cell lines upon exposure to different LXA4 concentrations (figure 4A and 4B). In both cell lines, significant responses were observed at concentrations as low as 1 pM and the maximum response achieved at 10 nM LXA_4_ (Figure 4C).

As reported in Table 1, the inward and outward conductances were significantly increased by LXA_4_ (100 nM, 10 min) without any change in the reversal potential in all cell types studied including primary cultures of CF and non-CF bronchial epithelia and CF and non-CF cell lines.

### Role of the ALX/FPR2 Receptor in the Whole Cell Conductance Responses to LXA_4_

The inhibitory effect of the Boc-2 antagonist on calcium responses to LXA_4_ indicate a role for the ALX/FPR2 receptor in transducing LXA_4_ responses in airway epithelial cells. This is also true for lipoxin effects on membrane ionic currents where treatment of Nuli-1 and CuFi-3 cells with Boc-2 (10 µM) completely abolished the stimulatory effect of LXA_4_ on the whole-cell current. As indicated in figure 4C, Boc-2 did not affect the basal inward conductance but significantly prevented the increase in inward conductance induced by LXA_4_ (Figure 4C).

### Role of Intracellular Ca^2+^ in the Whole-cell Conductance Responses to LXA_4_

The role of intracellular Ca^2+^ in the LXA_4_ induced whole-cell currents was evaluated using BAPTA-AM in the patch pipette as a chelator of intracellular Ca^2+^. Under these conditions of low intracellular Ca^2+^, the basal whole-cell conductances remained unchanged, whereas the lipoxin-stimulated currents were absent in BAPTAM-treated cell. The use of BAPTA-AM demonstrated the absolute requirement for an increase in intracellular calcium levels to transduce the effect of LXA_4_ on whole-cell inward conductance in both NuLi-1 cells and in CuFi-3 cells (Figure 4C).

### LXA_4_ Effect on Cl^−^ Secretion

**Figure 5:**
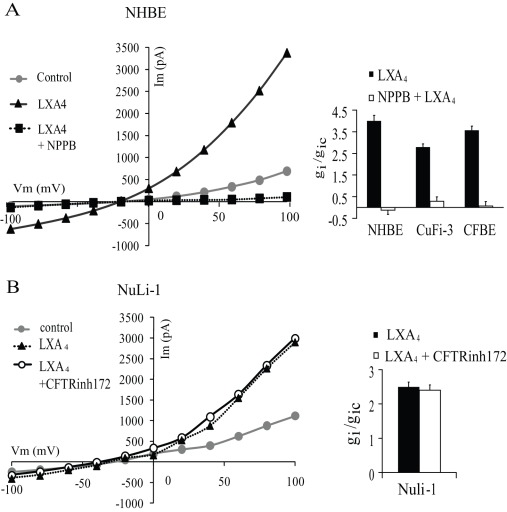
Effects of Cl^−^ channel inhibitors on whole-cell currents stimulated by LXA_4_. (A) Representative I-V relationship of the effect of NPPB (1 µM) after the stimulation of primary cultures of bronchial epithelial cells with LXA_4_ (100 nM). Corresponding histogram of the inhibitory effect of NPPB on the inward conductance obtained in non-CF primary NHBE cells (n = 4), CuFi-3 cells (n = 3) and CF primary CFBE cells (n = 3) after stimulation with LXA_4_ (100 nM, 10 min). (B). Typical I-V relationships of the effect of LXA_4_ (100 nM) obtained in NuLi-1 cells after treatment with the CFTR channel inhibition CFTR-inh172 (5 mM) and histogram showing the absence of inhibitory effect in Nuli-1 cells (n = 3).

The contribution of Cl^−^ channels to the LXA_4_ induced current, was tested using chloride ion channel inhibitors; NPPB (calcium-activated Cl^−^ channel inhibitor) and CFTR-inh172 (CFTR Cl^−^ channel inhibitor), and recording their effect on membrane current and conductance responses to LXA_4_ in CF and non-CF cells (Figure 5). Pre-treatment of CFBE primary cultured cells for 2 min with NPPB (1 µM) prior to LXA_4_ exposure, completely inhibited the stimulatory effect of LXA_4_ on whole-cell current (p>0.5, n = 3). Furthermore, the addition of NPPB (1 µM) 10 min after exposure to LXA_4_ (100 nM), immediately inhibited the whole-cell currents in non-CF and CF cells. Figure 5A illustrates a typical experiment performed in NHBE primary cells showing the inhibitory effect of NPPB treatment after LXA4 stimulation of the whole cell currents. A summary of the inhibitory effects of NPPB on inward conductance in the CF and non-CF primary cultures, and CF cell line (CuFi-3) are presented in figure 5A. As an additional proof for the role of Cl^−^ channels in driving the LXA_4_ induced current, we found that in the absence of an electrochemical driving force for chloride ions (equimolar Cl^−^ replacement in the bath and patch pipette), LXA_4_ did not produce a change in whole-cell current in any of the cell types tested (data not shown).

The specific CFTR inhibitor, CFTR-inh172, had no effect of CFTR-inh172 on basal or LXA_4_-stimulated membrane current and conductance in CFBE and CuFi-3 cells (data not shown), consistent with the absence of functional CFTR. Moreover, the CFTR inhibitor did not affect the stimulation of the whole-cell current by LXA_4_ in NuLi-1 cells (Figure 5B). When Nuli-1 cells were pre-treated with CFTR-inh172 (5 µM), subsequent LXA_4_ exposure stimulated the outward and inward conductances by 2.1±0.15 fold and 2.5±0.14 fold (n = 3), respectively (Figure 5B). These results indicate that the stimulatory effect of LXA_4_ on whole-cell current and conductance in non-CF and CF bronchial cells is mainly due to activation of NPPB-sensitive Cl^−^ channels and does not involve CFTR channels.

### LXA_4_ Effects on Airway Surface Liquid Height

**Figure 6:**
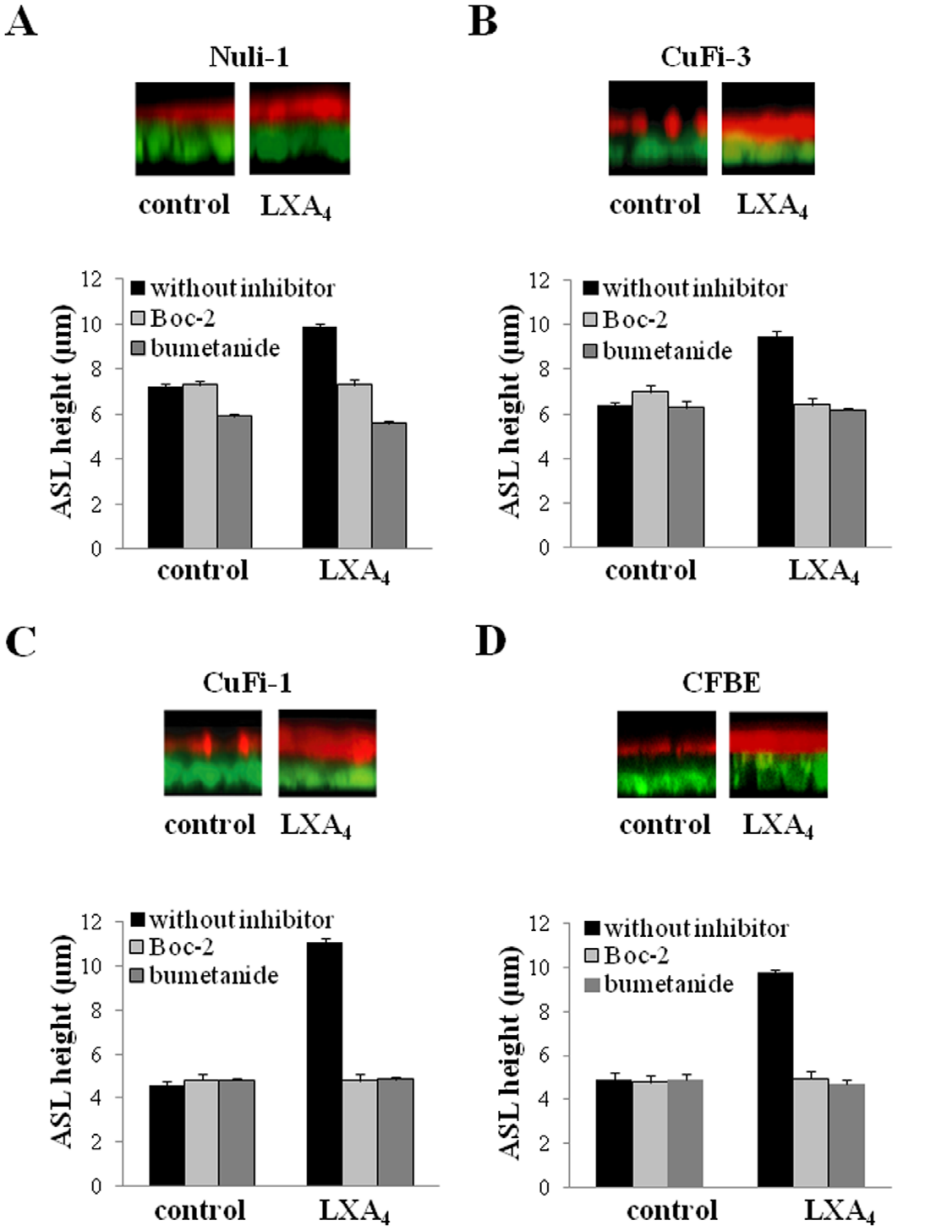
LXA_4_ (100 nM) effects on airway surface liquid height in NuLi-1 (A), CuFi-3 (B) and CuFi-1 epithelial cell line (C) and CFBE primary cultures (D). Epithelial cells were stained with calcein green, and the ASL labelled with dextran-conjugated Texas red™ fluorochrome. For each cell preparation, typical z-plane confocal sections showing ASL responses to LXA_4_ (top) and mean ASL height changes (bottom) in control conditions or following LXA_4_ (100 µM) exposure in NuLi-1,CuFi-3, CuFi-1 and CFBE epithelia treated or not with Boc-2 (10 µM) and with bumetanide (10 µM).

The consequence of LXA_4_ stimulation of Cl^−^ channels and transepithelial Cl^−^ secretion on ASL height was investigated in the cell lines (Nuli-1, CuFi-1 and CuFi-3) and in primary cultures of non-CF and CF airway epithelial cells grown on permeable supports under an air/liquid interface. The ASL measurements were carried out after a period of 24 hours exposure to apical fluid (8 µl of PBS) to allow fluid absorption/secretion and ASL height to reach a steady state. Under control conditions (without LXA_4_), the non-CF epithelia from NuLi-1 cell and primary cultures displayed a continuous unbroken ASL layer (*not shown*) whereas CF epithelia from cell lines and primary cultures presented a disrupted and thinner ASL layer (figure 6). Exposure to LXA_4_ (100 nM, 15 min) increased the ASL height in both non-CF and CF cell lines and primary cultures (figure 6). LXA_4_ treatment produced a maximum ASL height increase from 7.25±0.07 µm (n = 6) to 9.9±0.1 µm (n = 6) in Nuli-1 epithelia (p<0.001) and from 4.6±0.20 µm (n = 6) to 11.1±0.20 µm (n = 6) in CuFi-1 epithelia (p<0.001), and from 6.4±0.1 µm (n = 6) to 9.5±0.2 µm (n = 6) in CuFi-3 epithelia (p<0.001) and from 4.9±0.32 µm (n = 9) to 9.8±0.10 µm (n = 9) in CFBE primary cultures (p<0.001). The ASL height response to LXA_4_ obtained in CFBE primary cultures from 3 different CF patients was found to be robust and similar to the ASL response obtained in CuFi-1 differentiated cells. In all of the CF bronchial epithelia, the disrupted appearance of the ASL layer was absent following LXA_4_ treatment (Figure 6 B, C, D).

### Role of Cl^−^ Transport in the ASL Height Responses to LXA_4_

The Na/K/2Cl co-transporter inhibitor bumetanide was used to investigate the contribution of Cl^−^ secretion to the generation of the ASL. Bumetanide (1 µM) treatment significantly decreased the basal ASL height (control 7.25±0.07 µm, bumetanide 5.9±0.1 µm (p<0.001, n = 6)) in NuLi-1 monolayers but had no significant effect on ASL height in CuFi-1 epithelia (control 4.6±0.1 µm, bumetanide 4.82±0.10 µm (p>0. 5, n = 6)), in CuFi-3 epithelia (control 6.4±0.1 µm, bumetanide 6.3±0.3 µm (p>0.5, n = 6)) and in CFBE (control 4.9±0.32 µm, bumetanide 4.9±0.25 µm (p>0.5, n = 8)). These data indicate that Cl^−^ secretion contributes to the generation of the basal ASL height in the non-CF epithelium. Furthermore, bumetanide (1 µM) significantly abolished the ASL height increase induced by LXA_4_ in cell types tested. The ASL height measured after LXA_4_ exposure in the presence of bumetanide was significantly decreased in NuLi-1 (5.6±0.11 µm, n = 5, p<0.05), in CuFi-1 (4.86±0.1 µm, n = 6, p<0.001) and in CuFi-3 (6.2±0.1 µm, n = 6, p<0.001) cell lines and in CFBE primary cultures (4.7±0.2 µm, n = 9, p<0.001) compared to LXA_4_ alone (Figure 6). Taken together, these results demonstrate that the stimulatory effect of LXA_4_ on ASL height mainly involves Ca^2+^-dependent Cl^−^ secretion via NPPB-sensitive channels in both CF and non-CF epithelia grown from cell lines and primary cultures.

### Role of the ALX/FPR2 Receptor in the ASL Height Responses to LXA_4_

We tested the effect of the ALX/FPR2 receptor antagonist Boc-2 on the ASL height response to LXA_4_. Boc-2 significantly reduced the effect of LXA_4_ on ASL height in Nuli-1, CuFi-1 and CuFi-3 bronchial epithelial cell lines and CFBE primary cultures without affecting the basal ASL height (Figure 6). These data support the conclusion that the LXA_4_ receptor ALX/FPR2 mediates the effect of LXA_4_ on airway surface liquid height as well as on calcium mobilization and Cl^−^ secretion in bronchial epithelium.

## Discussion

This is the first study to report a novel effect of the endogenous lipoxin LXA_4_ to stimulate an increase in Airway Surface Liquid height, by enhancing Ca^2+^ activated Cl^−^ transport in bronchial epithelial cells obtained from patients with CF and non-CF patients and in airway cell lines.

In the healthy lung, the ASL forms a thin layer of fluid on the surface of the bronchial epithelium which allows cilia to beat effectively [8]. Maintenance of an optimal ASL height for ciliary beat is crucial for the efficacy of mucociliary clearance [8]–[12]. Bronchial epithelial ion transport regulates the ASL height, mainly by generating osmotic gradients which provide the driving force for transepithelial water movement [13]. In CF, the lack of functional CFTR leads to a reduced ASL height, resulting in an impaired mucociliary clearance that promotes chronic bacterial infection of the airways [3]. In a previous study, we reported that LXA_4_ stimulated an intracellular Ca^2+^ mobilization in a normal human airway epithelial cell line 16HBE14o- [7]. Here, we tested the hypothesis that LXA_4_ induces a Ca^2+^ signal to drive an increased Cl^−^ secretion and ASL height in CF epithelium. Lipoxin A_4_ thus stimulates a compensatory calcium-activated chloride secretory mechanism which overcomes the lack of CFTR mediated Cl^−^ transport and enhances airway lumen hydration.

Other studies have shown that LXA_4_ exerts biological actions on human airway epithelial cells, with a maximal effect observed at 100 nM. LXA_4_ (100 nM) inhibited IL-8 production by airway epithelial cells [14], stimulated an intracellular Ca^2+^ signaling [7], increased Z0–1 expression and transepithelial electrical resistance [15], and enhanced epithelial repair after an acid injury [16]. We report here, novel effects of LXA_4_ on ion and fluid transport in normal and CF bronchial epithelia.

Our studies show that LXA_4_ induces an intracellular Ca^2+^ mobilization in normal and CF epithelia. The Ca^2+^ response to LXA_4_ involves signal transduction via the ALX/FPR2 receptor, since the FPR2 receptor antagonist Boc-2 inhibited the LXA_4_ effect. This result supports our previous study which suggested that the Ca^2+^ signal induced by LXA_4_ was mediated by the ALX/FPR2 receptor since the Ca^2+^ response to LXA_4_ was only obtained in the 16HBE14o- airway epithelial cell line that express the receptor whereas LXA_4_ did not produce any Ca^2+^ response in the A549 cell line which does not express ALX/FPR2 [7].

Our results indicate that in non-CF airway epithelial cells, LXA_4_ generates a rapid and transient calcium signal mainly arising from the release of Ca^2+^ from intracellular stores and not as a result of increased Ca^2+^ entry since the calcium signal was not affected by the removal of external Ca^2+^. This is in agreement with our previous findings showing that the Ca^2+^ mobilization induced by LXA_4_ was generated from thapsigargin sensitive stores [7]. In contrast, in CF airway epithelial cells, the duration of the Ca^2+^ signal induced by LXA_4_ was greater than in non-CF cells. Although, in CF cells, the removal of external calcium did not affect the maximum peak calcium increase, the calcium response to LXA_4_ became more transient. These results suggest that, in CF airway epithelial cells, in addition to the calcium release from intracellular stores, LXA_4_ also stimulates calcium entry which leads to an overall larger calcium mobilisation than in normal airway epithelial cells. The observed differences reported in the literature between the Ca^2+^ signal obtained upon agonist exposure in CF and non-CF airway epithelial cells are controversial. Some authors reported that expression of either CFTR or ΔF508CFTR in airway epithelial cells had no effect on intracellular Ca^2+^ [17]. However, our results are in accordance with the demonstration that Ca^2+^ signaling is abnormal in CF airway epithelial cells and that correction of the abnormal trafficking of ΔF508CFTR protein restored intracellular Ca^2+^ homeostasis [18]. Recent reports also indicate that intracellular Ca^2+^ signals induced by pro-inflammatory mediators are increased in CF airway epithelia compared to non-CF due to an expansion of the apical endoplasmic reticulum Ca^2+^ stores in CF airway epithelial cells [19]. This finding is consistent with several studies showing that the nasal transepithelial electrical potential responses to agents that promote an intracellular Ca^2+^mobilization and Ca^2+^-dependent Cl^–^ conductance were higher in CF patients than in normal subjects [20]–[23].

Intracellular Ca^2+^ regulates several epithelial functions including ion transport, mucin secretion, and ciliary beat frequency which constitute a primary mode of a non-specific cleansing process and lung protection. Our results indicate that the Ca^2+^ signal induced by LXA_4_ is coupled to an increased Cl^−^ secretion in CF epithelium. LXA_4_ stimulated the whole-cell current and conductance in non-CF and CF epithelial cells. The inhibitory effect of BAPTA-AM used as a chelator of intracellular Ca^2+^ demonstrated the essential role of Ca^2+^ in the stimulation of the whole-cell currents by LXA_4_. The sensitivity of basal and stimulated whole-cell currents to NPPB or Cl^−^ substitution, underscores the major contribution of Cl^−^ secretion to the generation of the whole-cell current. These results agree with our previous report indicating that LXA_4_ stimulated a Ca^2+^-activated transepithelial Cl^−^ secretion in non-CF bronchial epithelial cells [7]. Since we found that LXA_4_ stimulation of the whole-cell currents was present in CF airway epithelia (in which CFTR is not functionally expressed), LXA_4_ most probably affects Cl^−^ channels other than CFTR. In addition, we found that the duration of the LXA_4_ effect on whole-cell currents was different between non-CF and CF cells, with a transient current increase in non-CF cells compared to CF cells where the current increase was more sustained. One explanation may be that the time course of the effect of LXA_4_ on the Cl^−^ currents is directly related to the time course of the intracellular calcium change induced by LXA_4_. Therefore, the greater and sustained effect of LXA_4_ on whole-cell currents in CF cells could be related to the long lasting Ca^2+^ signal obtained in CF airway epithelial cells. Finally, the ineffectiveness of CFTR inh-172 on the LXA_4_ stimulated whole-cell currents indicates that the effect of LXA_4_ on Cl^−^ secretion is not mediated by CFTR activation. This conclusion is strengthened by the observation that bumetanide reduces further the ASL height compared to Boc-2 treatment in Nuli-1 cells but not in CuFi-3 cells where functional CFTR is absent. If LXA_4_ had stimulated CFTR and Ca^2+^-dependent Cl^−^ channels together we would expect equivalent inhibition of ASL height by Boc-2 and bumetanide in Nuli-1 cells.

We have described a novel stimulatory effect of LXA_4_ on Cl^−^ secretion which produces an increased ASL height in both normal and CF epithelia. The CF bronchial epithelia generate a thinner ASL layer than non-CF airway epithelia. This finding is consistent with the diminished ASL in CF airways reported in the literature [12]. In addition, we observed that in control conditions, the non-CF epithelial monolayers showed a continuous ASL layer whereas in CuFi-1, CuFi-3 cell lines and CFBE primary cultures the liquid layer was disrupted. The gaps in the airway surface liquid layer result from localised de-hydration of the ASL. Following LXA_4_ exposure in CF bronchial epithelia, the ASL height significantly increased and appeared uniform. The inhibitory effect of bumetanide indicates that the effect of LXA_4_ on ASL height is mainly dependent on stimulation of transpithelial Cl^−^ transport. However, we cannot exclude the possibility that LXA_4_ can also exert its action to increase ASL height through the inhibition of ENaC activity which is known to be stimulated in CF airway and down-regulated by increased intracellular Ca^2+^.

An important outcome of this work is the comparable results found for LXA_4_ effects on ASL height in cell lines and primary CF cultures. We have demonstrated that using thin film airway liquid cell culture techniques, the NuLi and CuFi cell lines provide a robust model of airway liquid dynamics. Although ASL volume measurements have been published for the Calu-3 airway cell line [24], it is important to distinguish between ASL volume and height measurements and their meaning for effective mucociliary clearance. ASL height is the relevant physiological parameter as it determines the effective beating of cilia which must be covered to an optimal height with ASL. The volume of ASL on the other hand may change without revealing the true optimal ASL height covering the cilia but instead may reflect flooding of the airways which would also render cilia beat inefficient for mucocilary clearance. Although the use of primary airway epithelia tissue is the ideal for research in CF, the access to patient tissue samples, particularly children with CF and ‘normal’ non-CF controls as in our study, is non-trivial. The use of particular airway cell lines such as NuLi and CuFi which in our hands display normal and CF airway epithelium phenotype to secrete a thin ASL and mucus provides an additional validated cell model for CF research where human tissue samples are rare and difficult to obtain for research purposes.

Taken together, our results provide evidence for a novel role of LXA_4_ in stimulating Ca^2+^ activated Cl^−^ secretion and ASL generation in CF and non-CF airway epithelium. Thus LXA_4_ or its stable analogues may provide a novel therapeutic strategy to rehydrate the CF airway by modulating ion transport and airway surface liquid height via pathways which bypass defective CFTR. Our findings also indicate that the reduced levels of LXA_4_ observed in CF patients may be an additional contributory mechanism by which mucociliary clearance is diminished in CF airways.

## Materials and Methods

### Cell Culture

For the primary culture of human bronchial epithelium, the cells were obtained from bronchial brushing specimens obtained from 6 children (<6 years old) with CF and 5 non-CF controls through the SHIELD CF study (Study of Host Immunity and Early Lung Disease in CF). The children with CF were homozygous for the Phe508del mutation. Local ethics committee approval for the study was granted and written informed consent obtained. Bronchial epithelium brushings were washed and incubated for two hours at room temperature with 250 µg/ml amphotericin B in Phosphate Buffer Saline (PBS) without calcium and magnesium. After centrifugation, the pellet was collected and re-suspended in 500 µl of Bronchial Epithelium Basal Medium (BEBM, Clonetics, BioWhittaker, San Diego, USA) supplemented with 0.5 µg/ml human recombinant epidermal growth factor, 7.5 mg/ml bovine pituitary extract, 0.5 mg/ml epinephrine, 10 mg/ml transferrin, 5 mg/ml insulin, 0.1 µg/ml retinoic acid, 6.5 µg/ml triiodothyronine, and 50 mg/ml gentamicin (BD, Erembodegem, Belgium) and 250 µg/ml amphotericin B (BD, Erembodegem, Belgium). The explants were plated in a 24 well plate (Nunc, Roskilde, Denmark) previously coated with a fibronectin/collagen solution and incubated at 37°C in a humidified 5% CO_2_ atmosphere. Twenty four hours after seeding, the volume of media was adjusted to 400 µl. The cells were cultured under these conditions for six to nine days (confluence close to 70%) before splitting. Fibroblasts were removed by 1 minute treatment with trypsin EDTA (Gibco, Invitrogen, Paisley, UK). Epithelial cells referred in this paper as NHBE (non-CF bronchial epithelial cells) and CFBE (CF bronchial epithelial cells) were then trypsinised and re-suspended after centrifugation, in supplemented BEBM. The cells were seeded at 2500–4000 cells/cm^2^ in flasks (BD, Erembodegem, Belgium).

NuLi-1, CuFi-1, CuFi-3, and CuFi-4 cells were kindly donated by Prof Zabner,University of Iowa, USA. The NuLi-1 cell line was derived from human airway epithelium of normal genotype, whereas CuFi-1, Cufi-3 and CuFi-4 cell lines were derived from CF patients with Δ508/Δ508, R553X/Δ508, and G551D/Δ508 genotypes respectively. The cell lines were transformed with a RT component of telomerase and human papillomavirus type 16 E6 and E7 genes [25]. Cells were initially grown to confluency in flasks using BEBM with EGF, hydrocortisone, bovine pituitary extract, transferin, bovine insulin, triiodothyronine, epinephrine, retinoic acid, penicillin-streptomycin (0.025 µg/ml), gentamicin (0.05 ng/ml), and amphotericin (25 µg/ml).

Airway epithelial cells were plated at 2×10^6^ cells/cm^2^ on Millicell hanging cell culture inserts (Millipore, Billerica, USA) for ASL height measurements. All inserts were pre-coated with collagen type VI and grown in BEGM medium until confluence was achieved. Once cell confluence was confirmed under visual inspection, the medium was switched to DMEM/F-12 (Invitrogen, Auckland, New Zealand) to aid cell differentiation. This medium was supplemented with Ultroser G (2%, Pall Biospera, Cergy-Saint-Christophe, France), which enhances ion transport [25], and penicillin-streptomycin (0.025 µg/ml), gentamicin (0.05 ng/ml), and amphotericin (25 µg/ml). Medium at the apical aspect was aspirated every 3–4 days until the establishment of an air-liquid interface. The basolateral culture medium was replaced every 2–3 days. After 4–6 weeks growth, the cells formed a polarised confluent monolayer with a high transepithelial electrical resistance (TER) of >700 Ω/cm^2^.

### Intracellular Calcium Imaging

Intracellular Ca^2+^ was measured by epifluorescence microscopy as previously described [26]. The human airway epithelial cells were cultured on fibronectin-collagen coated (for primary culture cells) and on collagen VI coated (for NuLi-1 and CuFi-1 cells) glass bottom dishes (WPI, Stevenage, UK) for 6 days until 70% of confluence was reached. Cells were loaded with 5 µM of the Ca^2+^ -sensitive fluorescent probe fura-2-acetoxy-methyl ester (fura 2-AM, Invitrogen, Auckland, New Zealand) for 30 min, in the dark, at room temperature (22°C) and were then washed twice in HEPES-buffered Krebs-Henseleit solution (140 mM NaCl, 5 mM KCl, 2 mM CaCl2, 1 mM MgCl_2_, 10 mM HEPES, pH 7.4, 280–290 mOsmol). The glass bottom dishes covered with the fura 2-AM loaded epithelial cell monolayer were mounted on the stage of an inverted microscope equipped for epi-fluorescence (TE-300, Nikon, Badhoeve Dorp, Netherlands). Intracellular Ca^2+^ imaging was performed using the Metafluor Imaging System (Universal Imaging Corporation). The cell preparation was excited alternatively with monochromatic light at 340 and 380 nm using an Optoscan monochromator (Cairn Research Ltd, Kent, UK). The emission fluorescence produced after fura 2-AM excitation was filtered at 512 nm. The emitted light image was detected using a Photometrics CoolSNAP-fx video camera (Roper Scientific, Evry, France) coupled to the microscope. The fluorescence obtained at each excitation wavelength (F340 and F380) depended upon the level of Ca^2+^ binding to fura 2-AM. The results are given as ratiometric data (F340/F380), or as amplitude of variation compared to the basal ratio level (ΔF340/F380).

### Whole-cell Patch-clamp Recording

Freshly isolated epithelial cells obtained from 4 non-CF patients and from 3 CF patients (genotype: ΔF508/ΔF508) and from the NuLi-1 and CuFi-3 cell lines were used for patch-clamp experiments. The CuFi-1 cells were not used for patch-clamp experiments since we could not reach a Giga ohm seal with these cells. Cells were patch-clamped at room temperature (25°C) on an inverted microscope (TE-300, Nikon, Badhoeve Dorp, Netherlands). Patch-pipettes were prepared from soda glass (Vitrex, Modulhom, Herlev, Denmark), pulled on a programmable puller (P80/PC, Sutter Instrument Company, USA). The whole-cell configuration was obtained from cell-attached mode after breaking the patch membrane by applying a brief negative pressure in the patch pipette. Whole-cell currents were amplified (Axopatch 200B, Axon instrument, CA) and digitized using a 16-bit data converter (Digidata 1322A, Axon instrument, CA) following low pass filtering at 5 Khz and sampled in real-time. Whole-cell current voltage (IV) relationships were analysed using Clampfit software (Axon instrument, CA).

The patch pipette was filled with a “high K^+^ solution” at pH = 7.2, 290 mosm: 110 mM K-gluconate, 20 mM NaCl, 1.2 mM KH_2_PO_4_, 3.46 mM, 3mM KH_2_PO_4_, 5 mM EGTA, 6 mM HEPES, 2.78 mM CaCl2, pH = 7.2 adjusted with KOH. The bathing solution had the following composition: 140 mM NaCl, 5 mM KCl, 6 mM Hepes, 2 mM CaCl_2_, 1.2 mM KH_2_PO_4_, 1.2 mM MgSO_4_ and pH = 7.4. The Nernst potentials between the patch pipette and bath for K^+^ and for Cl^−^ were −77 mV and −39 mV, respectively. The access resistance (*R*a) was determined by fitting the current transients produced by a 5mV voltage pulse with a single exponential. The measured *R*a was 5.43±0.08Ω (n = 40).

For experiments performed in “low internal calcium”, cells were bathed in Kreb’s solution and the patch pipette contained 100 nM CaCl_2_ with 5 mM EGTA and 10 µM BAPTA-AM. In these conditions the free Ca^2+^ has been estimated at 1 pM using the free software WEBMAXC http://www.stanford.edu/~cpatton/webmaxc/webmaxcS.htm.

### Airway Surface Liquid (ASL) Height Measurements

ASL height was measured using a protocol adapted from Tarran *et al*. (10), using live-cell confocal fluorescence microscopy. To label the ASL, 8 µl PBS containing 1 mg/ml Texas red®-dextran (10 kD; Invitrogen, Auckland, New Zealand) was added to the apical surface of the well-differentiated airway epithelium. The epithelial cells were stained using Calcein-AM (5 µM, Invitrogen, Auckland, New Zealand) dissolved in medium culture for 30 minutes and introduced to the basolateral compartment of the insert. The Fluorinet™ electronic fluid Perfluorocarbon-72 (FC-72, 3M, St Paul, USA) was added to the apical compartment of the insert at a volume of 0.5 ml. Perfluorocarbon-72 is immiscible with the ASL and was used to prevent ASL evaporation on transferring the inserts from the incubator to the microscope stage and during the confocal scanning experiments. Epithelia were Z-scanned using a Zeiss LSM 510 Meta using a 40X objective. For each culture insert, 3 different microscope fields randomly chosen were XZ scanned. In each microscope field, the ASL height was measured using the Zeiss LSM Image analyser software (Carl Zeiss Microlmaging GmbH, Germany) in 9 separate regions randomly determined and then averaged. This method of quantification was carried out after blind analysis performed by multiple users. The n values referred to the number of culture inserts tested in a given condition.

### Drugs

The lipoxin LXA_4_ was purchased from Calbiochem. Aliquots of LXA_4_ solution (100 µM) in ethanol were stored at −80°C to avoid degradation of the molecule. The peptide Boc-Phe-Leu-Phe-Leu-Phe (Boc-2) (Phoenyx pharmaceutical, Belmont, USA) was used as specific inhibitor of the ALX/FPR2 receptor (11). For these latter experiments, cells were pre-incubated with 10^−5^ M Boc-2 for 24 hours at 37°C. BAPTA-AM (10 µM, Molecular probes, Leiden, Netherlands) was used to chelate intracellular Ca^2+^ (12). The 5-Nitro-2-(3-phenylpropylamino) benzoic acid (NPPB, Sigma, USA) used at 1 µM is an inhibitor of Ca^2+^-activated Cl^−^ channels (13). CFTRinh-172 an antagonist of the CFTR channel and bumetanide an inhibitor of the NKCC1 co-transporter were supplied by Sigma (14).

### Data Analysis

The intracellular Ca^2+^ variations were measured as the difference between the mean F340/F380 ratio during the 2 min prior to exposure to LXA_4_ and the ratio measured at the peak of the Ca^2+^ response and 2 and 5 min after the peak. In each experiment, the mean ratio was obtained from all cells in the microscopic field. In whole-cell patch-clamp experiments, conductances were determined by linear regression of the current-voltage relationship obtained in *n* cells. For the ASL height measurements, three confocal image acquisitions were performed on each culture insert and nine regions of interest were analysed in the acquisition field using the LSM image browser (Zeiss). Values were loaded in an Excel spreadsheet and averaged. Mean value were obtained from *n* independent experiments. The experiments were repeated under the same conditions on a minimum of three different cell passages. Data are presented as the mean ± S.E.M. of *n* experiments. Measures of statistical significance were obtained using the Student’s *t* test for paired data. A *p* value <0.05 was deemed to be significant. All statistical operations were performed using Excel software (Microsoft).
